# Dynamic role of personality in explaining COVID-19 vaccine hesitancy and refusal

**DOI:** 10.3389/fpsyg.2023.1163570

**Published:** 2023-06-15

**Authors:** Melissa N. Baker, Eric Merkley

**Affiliations:** ^1^Department of Political Science and Public Administration, University of Texas at El Paso, El Paso, TX, United States; ^2^Department of Political Science, University of Toronto, Toronto, ON, Canada

**Keywords:** vaccine hesitancy, vaccine refusal, anti-vaccination, personality, personality traits, COVID-19

## Abstract

Vaccine hesitancy and refusal are threats to sufficient response to the COVID-19 pandemic and public health efforts more broadly. We focus on personal characteristics, specifically personality, to explain what types of people are resistant to COVID-19 vaccination and how the influence of these traits changed as circumstances surrounding the COVID-19 pandemic evolved. We use a large survey of over 40,000 Canadians between November 2020 and July 2021 to examine the relationship between personality and vaccine hesitancy and refusal. We find that all five facets of the Big-5 (openness to experience, conscientiousness, extraversion, agreeableness, and negative emotionality) are associated with COVID-19 vaccine refusal. Three facets (agreeableness, conscientiousness, and openness) tended to decline in importance as the vaccination rate and COVID-19 cases grew. Two facets (extraversion and negative emotionality) maintained or increased in their importance as pandemic circumstances changed. This study highlights the influence of personal characteristics on vaccine hesitancy and refusal and the need for additional study on foundational explanations of these behaviors. It calls for additional research on the dynamics of personal characteristics in explaining vaccine hesitancy and refusal. The influence of personality may not be immutable.

## 1. Introduction

What types of people are vaccine-hesitant? Research on the personal characteristics related to vaccine hesitancy has largely focused on traits such as political ideology and political knowledge. In a new and constantly changing environment, such as the COVID-19 pandemic, it is important to understand the role of more foundational personal traits, as the partisan and political implications of COVID-19 are fluid and context-dependent. We focus on personality traits, specifically the Big-5, to help explain why some people are more hesitant about vaccines than others.

Using a uniquely large, representative, multiwave survey of Canadians from November 2020 to July 2021, we find that the facets of the Big-5 (openness to experience, conscientiousness, extraversion, agreeableness, and negative emotionality) help explain the difference between vaccine accepters and refusers. The extended timeframe of our survey fielding also allows us to evaluate how these associations evolved with the pandemic, which has been missing from prior study on the influence of individual differences on protective behaviors during the COVID-19 pandemic. Our analyses show that the effects of personality on public health decisions are not always immutable, many of these effects changed with pandemic conditions.

### 1.1. Vaccine hesitancy and COVID-19

Research on the psychological foundations of vaccine hesitancy conducted before the COVID-19 pandemic shows that conservatives are more vaccine hesitant than liberals (Baumgaertner et al., 2018). These attitudes appear to translate to party identification, as Republicans in the United States are less likely to hold accurate beliefs about vaccines than Democrats (Joslyn and Sylvester, 2019; Motta, 2021). People with less political knowledge tend to have anti-vaccine policy preferences and believe they know more than vaccine experts (Motta et al., 2018). Relatedly, the expression of support for vaccine misinformation is to distrust of scientific experts (Stecula et al., 2020).

Vaccine hesitancy research related to the COVID-19 pandemic has largely focused on political ideology as an explanation for why some people are hesitant to receive a vaccine. In the United States, conservatism is related to greater COVID-19 vaccine hesitancy (Callaghan et al., 2021). These results are consistent with findings related to other public health guidelines, as conservatives are typically less likely to wear masks and engage in social distancing (Allcott et al., 2020; Gadarian et al., 2021).

### 1.2. Personality traits and COVID-19 vaccine hesitancy and refusal

Big-5 personality traits are important for a wide range of political attitudes and behaviors. This may also be true in relation to the COVID-19 pandemic. Our strongest expectation is an association between agreeableness and vaccine acceptance. Individuals who are more caring and considerate should be the most willing to vaccinate—an effort to protect other, oftentimes more vulnerable people from infection and harm. We have likewise strong expectations for conscientiousness. Individuals who are inclined toward diligence and rule-following are likely more inclined to follow public health recommendations regarding vaccination.

We expect the link between acceptance and two other Big-5 traits to be somewhat weaker. Openness to experience is likely to be associated with vaccination acceptance, perhaps due to the polarized debate surrounding COVID-19 vaccination. This is a trait that has been linked to left-wing ideological values in political psychological research (McCrae and Costa Jr, 1997; Van Hiel et al., 2000; Gerber et al., 2010). Openness could generate vaccination acceptance through that causal pathway. We anticipate a connection between negative emotionality and vaccine acceptance. Negative emotionality may make people disproportionately worried about the pandemic and incentivize them to vaccinate to protect themselves. We do not have any strong expectations related to the association between extraversion and vaccination acceptance.

This discussion leads us to the following hypothesis:

H1: agreeableness (A), conscientiousness (B), openness (C), and negative emotionality (D) are associated with lower levels of vaccine hesitancy and refusal.

Table 1 shows that these expectations are generally borne out by prior studies examining the connection between personality and COVID-19 precautionary behaviors. There is a consistently positive association between these actions and two traits: agreeableness and conscientiousness. Positive associations have also generally been found with openness and negative emotionality, though null results are also relatively common. Findings for extraversion have been wildly inconsistent in both their statistical significance and direction.

**Table 1:** Prior literature on the relationship between Big-5 personality traits and precautionary behavior.

**Study**	**Agreeableness**	**Conscientious**	**Openness**	**Negative emotionality**	**Extraversion**
Aschwanden et al. (2020)	+	+	+	–	+
Blagov (2021)	+	+	Null	+	–
Brouard et al. (2020)	+	+	Null	Null	–
Carvalho et al. (2020)	N/A	+	N/A	N/A	–
Chan et al. (2021)	+	Null	Mixed	Null	–
Clark et al. (2020)	Null	+	+	Null	–
Qian and Yahara (2020)	+	+	+	+	+
Rammstedt et al. (2022)	+	Null	Null	+	+
Volk et al. (2021)	Null	+	Null	+	+
Zajenkowski et al. (2020)	+	Null	Null	Null	Null
Zettler et al. (2022)	+	+	+	Null	+

There has been less research on the connection between COVID-19 vaccination and personality. The studies that do exist are generally inconsistent in their findings. Reagu et al. (2023) find positive associations between vaccine hesitancy and negative emotionality and openness in an opt-in convenience sample of Qatar residents recruited through online advertising. They also find a surprising positive association with conscientiousness. Nanteer-Oteng et al. (2022) find null results for personality traits in an opt-in social media-recruited convenience sample, while Lin and Wang (2020), who look at general attitudes toward vaccines rather than COVID-19 specifically, find positive relationships between vaccine acceptance and two traits—agreeableness and conscientiousness—and a negative relationship with negative emotionality.

This study will contribute to the existing literature by leveraging large, representative samples on this research question. The time frame of our fielding will also allow us to examine whether personality-driven differences in vaccination acceptance vary as pandemic conditions evolved between the end of 2020 and the first half of 2021. Expectations are not entirely obvious. On the one hand, we might see personality effects strengthen if the vaccine rollout campaign was the most successful at bringing on board those who are generally sympathetic toward vaccination (e.g., the agreeable), while others resisted. Similarly, these individuals might be most responsive to heightened risk as caseloads change. This story would be consistent with a motivated reasoning process.

On the other hand, it is possible that personality differences could shrink as the vaccination rate and cases rise. In this case, personality groups sympathetic to vaccination always intended to vaccinate, while the vaccine rollout campaign and changing epidemiological conditions were necessary to persuade everyone else. We note these general hypotheses below.

H2: The effects of personality traits grow as the vaccination rate (A) and COVID-19 cases (B) increase.
H3: The effects of personality traits weaken as the vaccination rate (A) and COVID-19 cases (B) increase.

## 2. Methods

To evaluate the relationship between Big-5 personality traits and vaccination intention we use surveys of Canadian adult citizens taken between November 2020 and July 2021 by the Media Ecosystem Observatory using the online sample provider Dynata. These 22 consecutive survey waves contained measures of Big-5 personality traits and reached 42,196 respondents. Canada offers a useful case for the study of COVID-19 behaviors, representing a middle ground between European and American (United States) experiences with the pandemic: relatively low levels of pandemic polarization among elites and the mass public (Merkley et al., 2020) and higher levels of trust in public health authorities, despite saturation by the United States news media and close cultural and social ties to that country (Bridgman et al., 2021).

Our fielding coincides with the rollout of the primary COVID-19 vaccine series in Canada, starting in January 2021 for healthcare workers and those in long-term care homes, expanding to older people in March and the rest of the population in April–May. It stops before provinces and the federal government established vaccine mandates and passports. It also coincides with the rise and fall of the Delta wave during late 2020 and early 2021, which became the dominant variant by the Summer of 2021. We are thus able to observe how the effects of personality vary with substantial changes in the COVID-19 pandemic in Canada.

Each wave used quotas to ensure the final sample matched population benchmarks on gender, region (Atlantic Canada, Quebec, Ontario, and West), age (18/34, 35/54, and 55+), and language (English and French). We use raked weighting to ensure gender and age balance within regions as well (maximum = 1.62, minimum = 0.73, and SD = 0.17).

### 2.1. Outcome

Our principle outcome measure is a question on vaccine intention “Would you take a vaccine to prevent COVID-19 infection once it becomes available?” (yes, no, and unsure). As the vaccine rollout began we included an option for “I have already been vaccinated.” These respondents are coded together with that report “Yes.” We label those in that category as *Accepters*, those who are uncertain as *Hesitant*, and those who report “No” as *Refusers*. Over our period of fielding the share of Canadian adults classified as *Accepters* increased from 62 to 82%.

### 2.2. Personality traits

We use the Big Five Inventory-2 Short Form (BFI-2-S). This form asks respondents to evaluate their personality across 30 different dimensions, a validated shorter version of the BFI-2 (Soto and John, 2017). Our alpha reliability scores for our five domain scales range from 0.61 for openness to 0.80 for negative emotionality. Our personality trait scales are standardized in the cross-sectional analyses that follow for ease of interpretation.

### 2.3. Estimation

We first estimated a pooled model where we regress vaccine intention on our Big-5 personality traits, a series of control variables for respondent age, respondent education level, annual household income, gender, region, and wave fixed effects. More details on the measurement of our variables can be found in Supplementary Table S1. We use multinomial logistic regression, treating vaccine intention as a categorical variable with Accepters as the reference category. We present the risk ratios and predicted probabilities from the model estimates to provide a substantive interpretation of the effects we observe. Over the course of the MEO surveys, some respondents were able to retake the survey—sometimes by design. We purge the dataset of duplicate respondents after their first appearance, leaving us with a sample of 26,316 unique respondents for our pooled model. We use these models to test our principal hypotheses.

The long timeframe of our fielding allows us to examine dynamics in the effects of personality traits on vaccine intention as pandemic conditions change. We focus on changes in the vaccination rate and COVID-19 caseload. We acquired COVID-19 case data from the World Health Organization.^1^ While WHO data uses official data from a wide range of health ministries, it primarily reflects laboratory-confirmed cases. At the start of fielding on 22 November 2020, daily new COVID-19 cases increased from just under 5,000 to almost 8,800 by 10 January 2021. They then fell to almost 2,500 by 4 March before rising yet again to over 9,500 on 17 April 2021. Cases stayed above 8,000 per day until falling to under 1,000 by mid-June.

We use vaccination rate data from Our World in Data.^2^ At the start of fielding no Canadians were vaccinated. The rate stayed at a trivial level until March 2021 when vaccinations opened to vulnerable populations such as the elderly. Between March and April 2021, the vaccination rate increased from 3% to almost one-third. Between May and June 2021, the rate further increased to over two-thirds.

The variables are common to respondents on the same day, so these respondents are nested within those days. We use multilevel modeling where we treat survey wave as the level variable because of an unequal daily rollout of the sample. Some days have systematically fewer respondents, usually from harder-to-reach quota groups. We thus use a wave-based average of our level 2 variables. We estimate two models per personality trait. The first interacts a given personality trait with the vaccination rate. The second adds an interaction of that trait with COVID-19 cases. We estimate the effect of the caseload interaction in the presence of the interaction with the vaccination rate because we expect as the rate increases, cases drop. The slope of the personality trait is allowed to randomly vary across groups. We cluster standard errors by wave.

## 3. Results

Table 2 presents the risk ratios from the pooled model estimates. We see only weak to non-existent associations between personality traits and the tendency to be a vaccine accepter vs. hesitant. One standard deviation (SD) increase in agreeableness is associated with a 9% increase in the relative risk of being hesitant compared to an accepter (*p* < 0.001). This supports H1A, though with a small effect size. A one SD increase in conscientiousness is also associated with a 5% increase in the relative risk of being hesitant (*p* < 0.05)—a small effect contrary to expectations for H1B. Ultimately, personality traits do not distinguish the vaccine-hesitant from accepters.

**Table 2:** Multinomial logistic regression estimates.

	**Refuser (vs. accept)**		**Hesitant (vs. accept)**	
	**Risk ratio**	**SE**	**Risk ratio**	**SE**
Negative emotionality	0.810^***^	0.022	1.047	0.027
Conscientiousness	0.903^***^	0.024	1.054^*^	0.028
Agreeableness	0.716^***^	0.018	0.910^***^	0.023
Extraversion	1.175^***^	0.028	1.026	0.024
Openness	0.894^***^	0.020	0.985	0.022
Respondent education level	0.885^***^	0.009	0.922^***^	0.010
Household income	0.882^***^	0.012	0.864^***^	0.013
Respondent age	0.965^***^	0.001	0.978^***^	0.001
Female	1.235^***^	0.054	1.295^***^	0.059
Quebec (reference = Atlantic)	1.091	0.099	0.874	0.078
Ontario (reference = Atlantic)	1.144	0.098	0.999	0.084
West (reference = Atlantic)	1.020	0.090	0.899	0.077
*N*	32,277			

Relative risk ratios, fixed effects not displayed.

^*^p < 0.05.

^**^p < 0.01.

^***^p < 0.001.

There are much stronger effects in the contrast between refusers and accepters. The strongest effect, by far, is with agreeableness, in support of H1A. A one SD increase in this measure is associated with a 28% reduction in the relative risk of being a vaccine refuser compared to an accepter (*p* < 0.001). There are also 10%, 11%, and 18% reductions in the relative risk of being a vaccine refuser with similar increases in conscientiousness, openness, and negative emotionality (in support of H1B-D; all significant at *p* < 0.001). We did not anticipate a significant effect of extraversion, but it appears to be associated with vaccine refusal—increasing its relative risk by 18%.

We provide the predicted probabilities from this model in Supplementary Figure S1. In the left panel, we see that moving across the agreeableness index (hereafter moving from −2 to +2 standard deviations) results in a decrease (increase) in the probability of being a vaccine refuser (accepter) from 0.23 (0.64) to 0.08 (0.80). In the top-center panel, we see moving across the openness index is associated with a decrease (increase) in the probability of being a vaccine refuser (accepter) from a more modest 0.17 (0.70) to 0.12 (0.75). The results are virtually identical for conscientious (top-right) while moving across the negative emotionality index is associated with a decrease (increase) from 0.21 (0.68) to 0.11 (0.75) in the probability of being a vaccine refuser (accepter). In contrast, moving across the extraversion index is associated with an increase (decrease) in the probability of being a vaccine refuser (accepter) from 0.11 (0.76) to 0.19 (0.69).

### 3.1. Multilevel results

**Figure 1:**
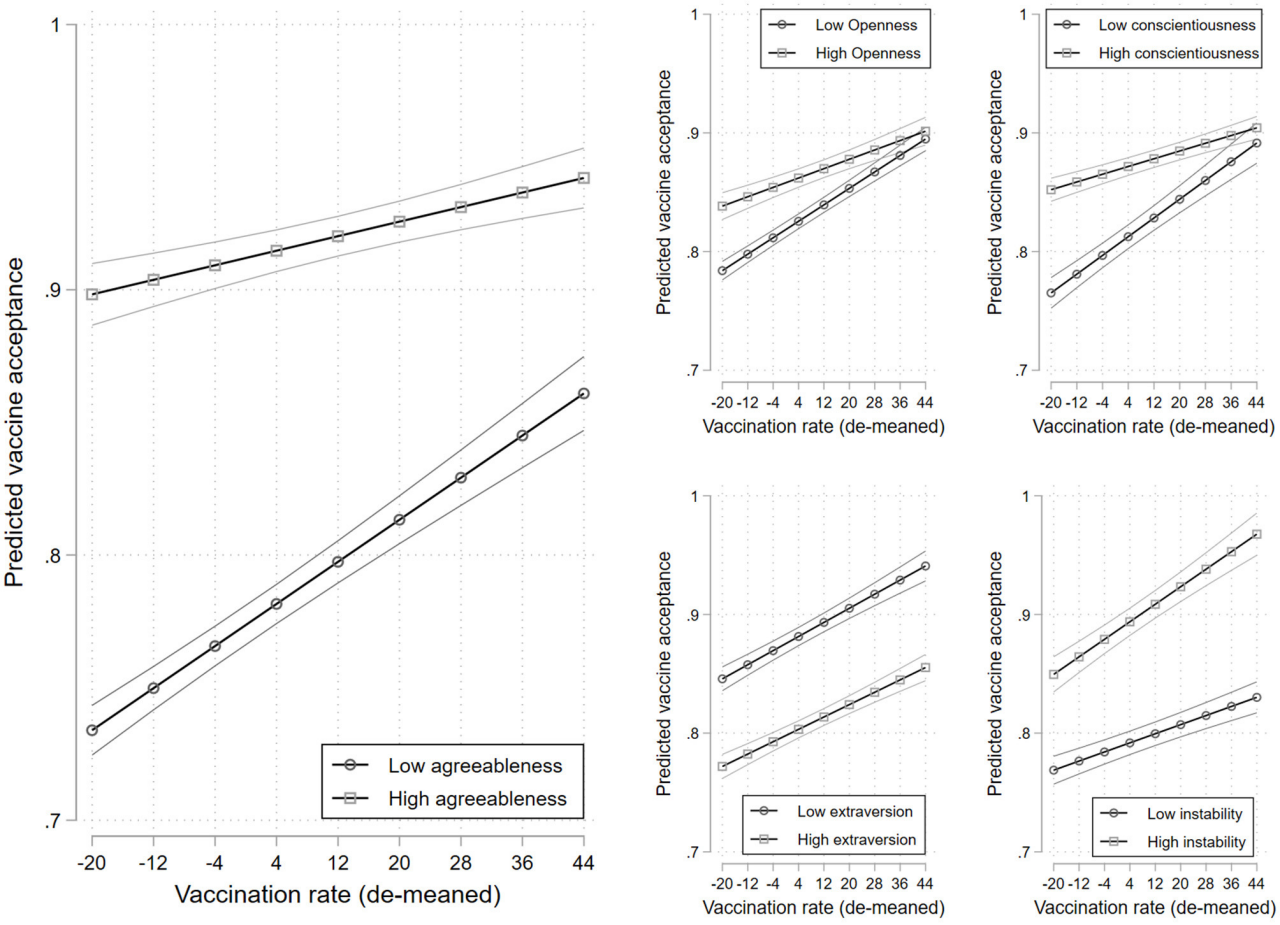
Predicted vaccine acceptance across levels of the aggregate vaccination rate for those with high and low levels of agreeableness **(left panel)**, openness **(top-center panel)**, conscientiousness **(top-right panel)**, extraversion **(bottom-center panel)**, and negative emotionality **(bottom-right panel)**. 95% confidence intervals. Estimates are provided in Supplementary Table S2.

There appears to be some dynamism in the effects of some of the Big-5 personality traits based on the results of our multilevel models. Agreeableness, openness, and conscientiousness seem to matter less as the aggregate vaccination rate increases. At the 5th percentile of the vaccination rate, there is a 0.17 difference in the probability of being a vaccine accepter between those in the 5th and 95th percentile of agreeableness. This drops to a 0.08 difference at the 95th percentile of the vaccination rate. This is illustrated in the left panel of Figure 1. The interaction term is significant at the 0.01 level.

We see a similar pattern for openness and conscientiousness. At the 5th percentile of the vaccination rate, there is a 0.06 (0.09) difference in the probability of being a vaccine accepter between those with high and low levels of openness (conscientiousness), which drops to an insignificant 1 point difference at the 95th percentile of the vaccination rate for both traits. These interactions are both significant at the 0.001 level. For these three personality traits, the story seems to be that as the mass vaccination campaign rolled out, harder-to-reach groups were brought on board.

There is a different pattern for negative emotionality. At the 5th percentile of the vaccination rate, there is a 0.08 difference in the probability of being a vaccine accepter between those with high and low levels of negative emotionality. This increases to a 0.14-point difference at the 95th percentile of the vaccination rate. This interaction term is also significant at the 0.001 level. Those with high negative emotionality also seem to be brought on board by the mass vaccination campaign. In contrast, we observe no dynamics in the effect of extraversion on vaccine acceptance, at least across varying levels of the vaccination rate (*p* = 0.350).

**Figure 2:**
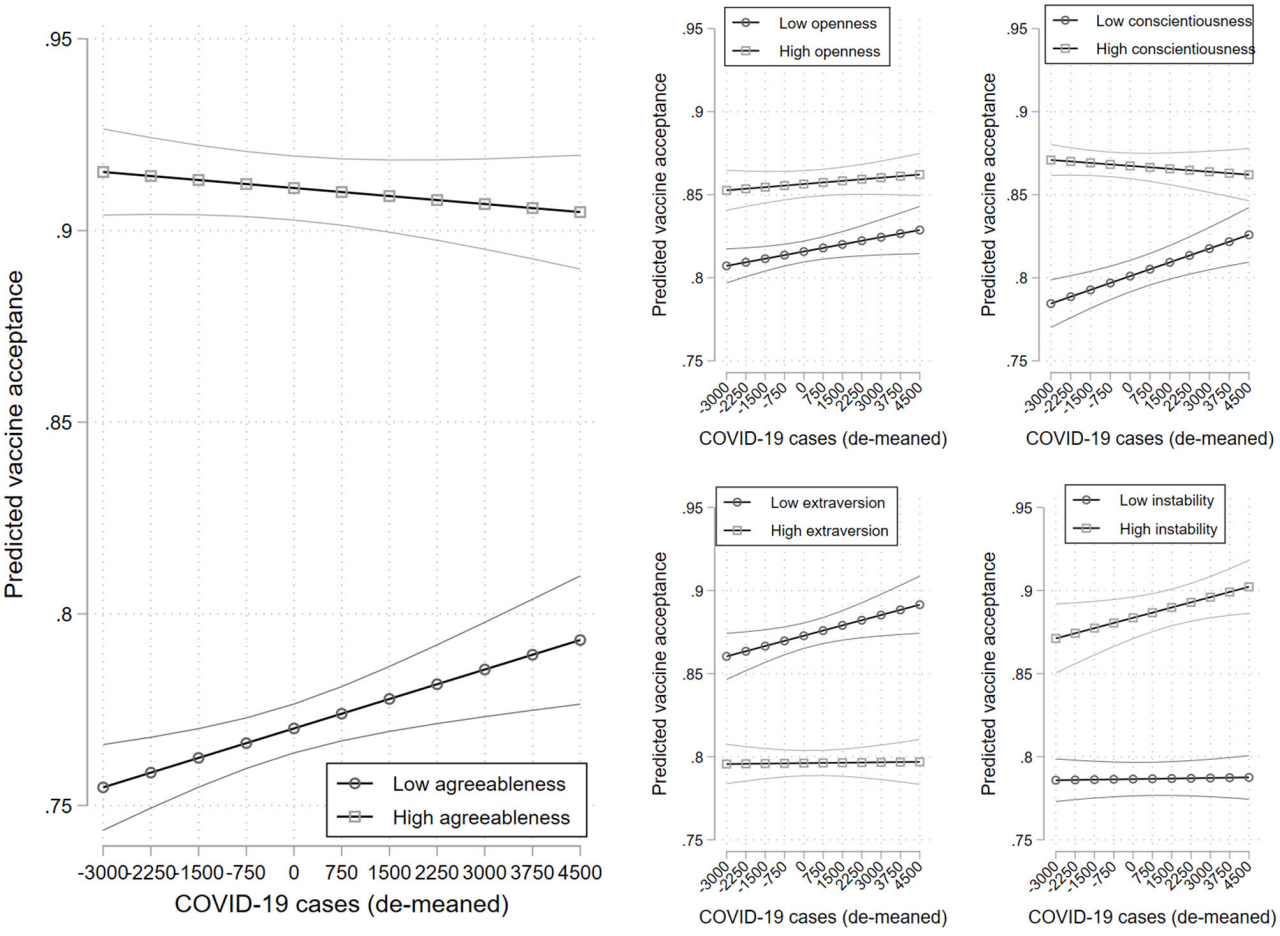
Predicted vaccine acceptance across levels of the aggregate COVID-19 caseload for those with high and low levels of agreeableness **(left panel)**, openness **(top-center panel)**, conscientiousness **(top-right panel)**, extraversion **(bottom-center panel)**, and negative emotionality **(bottom-right panel)**. 95% confidence intervals. Estimates are provided in Supplementary Table S3.

We see some dynamics across COVID-19 caseloads after controlling for the vaccination rate (Figure 2). Agreeableness and conscientiousness appear to matter less as cases rise. At the 5th percentile of cases, we see a 0.17 (0.09) difference in the probability of being a vaccine accepter between those with high and low levels of agreeableness (conscientiousness). This drops to a 0.11 (0.03) difference at the 95th percentile of the caseload. These interactions are significant at the 0.001 and 0.01 levels, respectively. There are no significant dynamics in the effects of openness, negative emotionality, or extraversion (*p* = 0.424; *p* = 0.138; *p* = 0.136), though there is a general pattern where the effects of emotional instability and extraversion grow as cases rise.

## 4. Discussion

Identifying who refuses COVID-19 vaccination is important for not only understanding the COVID-19 pandemic, but also for guiding future vaccination efforts related to novel viruses or persistent health threats like influenza. We find that all five facets of the Big-5 distinguish vaccine refusers from accepters. People who are lower in openness, conscientiousness, agreeableness, and negative emotionality are more likely to indicate they are not willing to receive a COVID-19 vaccine when it becomes available to them (consistent with H1A-D). These traits, however, do not consistently distinguish the hesitant from the accepters. The effects we observe here are modest, but still meaningful in comparison to other fundamental traits. An SD increase in our personality traits is associated with anywhere from an 11%−28% change in the relative risk of being a vaccine refuser. A similar increase in age, education, and income are associated with reductions of 49%, 25%, and 13% in this same risk, respectively.

Unexpectedly, people who are higher in extraversion are more likely to indicate they are not willing to receive a COVID-19 vaccine when it becomes available to them, a finding that persists regardless of the trajectory of COVID-19 cases or vaccination rates at the time. This is a peculiar finding deserving of further research. We might have expected extroverts to be more amenable to vaccination as a means of reducing the risk of social activity.

Openness, conscientiousness, and agreeableness matter less as a predictor of vaccine refusal as COVID-19 vaccination rates increase, and the same goes for the latter two traits as COVID-19 cases rise (consistent with H3). Negative emotionality, on the other hand, matters more as pandemic circumstances changed; people scoring high on this dimension are more likely to indicate a willingness to be vaccinated as vaccine rates increase (consistent with H2A). On balance, personality mattered less as vaccines rolled out and when risk escalated, but the story is not entirely consistent across personality traits.

Researchers, public health experts, and those responsible for policy implementation can use this information to inform future vaccination campaigns. For example, messaging strategies are often crafted in ways to appeal to different types of people. Our findings show that personality-based differences in vaccine refusal are not immutable, so it may be advantageous to work into these strategies. It appears that Canada's vaccine rollout failed to appeal to extroverts and those with low negative emotionality. We might expect the former to be persuaded by arguments that vaccines can help lower the risk of social activity and allow them to get back into the world and that the latter would not be so easily convinced by an emphasis on the risks of disease—other appeals may be needed.

Future research on vaccine hesitancy may wish to address ways in which personality interacts with other types of traits and circumstances to inform vaccine-related attitudes. For example, do strong personality effects persist when people are faced with social (e.g., familiar, occupational) pressure to get vaccinated? Does experiencing a mild or severe case of COVID-19 override personality effects that make people more susceptible to being open to or refusing vaccines? This research opens the door for much more research on the intricacies of how personality is related to vaccine hesitancy.

## Data availability statement

The raw data supporting the conclusions of this article will be made available by the authors, without undue reservation.

## Ethics statement

The studies involving human participants were reviewed and approved by University of Toronto Social Sciences, Humanities and Education Research Ethics Board. The patients/participants provided their written informed consent to participate in this study.

## Author contributions

MB and EM contributed to the conception and design of the study, collected the data, wrote the manuscript, contributed to the manuscript revision, read, and approved the submitted version. EM analyzed the data. All authors contributed to the article and approved the submitted version.

## Footnotes

^1^These data can be found here at https://covid19.who.int/data.

^2^These data can be found here at https://ourworldindata.org/covid-vaccinations.
