# Supplementary material for: Dynamic role of personality in explaining COVID-19 vaccine hesitancy and refusal
Source: Front Psychol. 2023 Jun 15;14:1163570. doi: 10.3389/fpsyg.2023.1163570 (PMC10311497; doi:10.3389/fpsyg.2023.1163570)
Supplement: Supplementary file 1 [file Data_Sheet_1.docx]

Supplementary Material

The Dynamic Role of Personality in Explaining COVID-19 Vaccine Hesitancy and Refusal

Melissa N. Baker*, Eric Merkley

*** Correspondence:** Corresponding Author: mnbaker@utep.edu

# Supplementary Tables

**Table S1.** Variable descriptions

| **Variable** | **Description** | **Mean** | **SD** | **Min** | **Max** |
| --- | --- | --- | --- | --- | --- |
| Vaccination intension | Would you take a vaccine to prevent COVID-19 infection once it becomes available? (0=No, 1=Unsure, 2=Yes/I have already been vaccinated) | 1.59 | 0.73 | 0 | 2 |
| Extraversion | 1) Tends to be quiet (reverse); 2) Is dominant, acts as a leader; 3) Is full of energy; 4) Is outgoing, sociable; 5) Prefers to have others take charge (reverse); 6) Is less active than other people (reverse); disagree strongly, disagree a little, neutral, agree a little, agree a lot; 0-1 scale | 0.51 | 0.17 | 0 | 1 |
| Neuroticism | 1) Worries a lot; 2) Tends to feel depressed, blue; 3) Is emotionally stable, not easily upset (reverse); 4) Is relaxed, handles stress well (reverse); 5) Feels secure, comfortable with self (reverse); 6) Is temperamental, gets emotional easily; disagree strongly, disagree a little, neutral, agree a little, agree a lot; 0-1 scale | 0.41 | 0.20 | 0 | 1 |
| Conscientiousness | 1) Tends to be disorganized (reverse); 2) Has difficulty getting started on tasks (reverse); 3) Is reliable, can always be counted on; 4) Keeps things neat and tidy; 5) Is persistent, works until the task is finished; 6) Can be somewhat careless (reverse); disagree strongly, disagree a little, neutral, agree a little, agree a lot; 0-1 scale | 0.68 | 0.18 | 0 | 1 |
| Openness | 1) Is fascinated by art, music, or literature; 2) Has little interest in abstract ideas (reverse); 3) Is original, comes up with new ideas; 4) Has few artistic interests (reverse); 5) Is complex, a deep thinker; 6) Has little creativity (reverse); disagree strongly, disagree a little, neutral, agree a little, agree a lot; 0-1 scale | 0.59 | 0.16 | 0 | 1 |
| Agreeableness | 1) Is compassionate, has a soft heart; 2) Is sometimes rude to others (reverse); 3) Assumes the best about people; 4) Can be cold and uncaring (reverse); 5) Is respectful, treats others with respect; 6) Tends to find fault with others (reverse); disagree strongly, disagree a little, neutral, agree a little, agree a lot; 0-1 scale | 0.67 | 0.17 | 0 | 1 |
| Education | 1) No schooling; 2) Some elementary school; 3) Completed elementary school; 4) Some secondary/high school; 5) Completed secondary/high school; 6) Some technical, community college, CEGEP, College Classique; 7) Completed technical, community college, CEGEP, College Classique; 8) Some university; 9) Bachelor's degree; 10) Master's degree; 11) Professional degree or doctorate; 12) Don't know (excluded) | 7.2 | 2.0 | 1 | 11 |
| Income | Total household income: 1) None; 2) $1-30,000; 3) $30,001-60,000; 4) $60,001-90,000; 5) $90,001-110,000; 6) $110,001-150,000; 7) $150,001-200,000; 8) More than $200,000; 9) Don't know, prefer not to answer (excluded) | 3.1 | 1.7 | 0 | 7 |
| Age | In years | 48 | 16 | 18 | 100 |
| Female | 1=female; 0=otherwise | 0.52 | 0.50 | 0 | 1 |
| Region | 1=Atlantic; 2=Quebec; 3=Ontario; 4=West | 3 | 0.9 | 1 | 4 |
| Vaccination Rate | Number of COVID-19 vaccines per 100 Canadians | 20.9 | 23.1 | 0 | 68.1 |
| COVID-19 Cases | Number of daily COVID-19 cases | 4175 | 2168 | 505 | 9570 |

**Table S2.** Multi-level model estimates, vaccination rate

|  | Agreeableness | Openness | Conscientiousness | Extraversion | Emotional instability |
| --- | --- | --- | --- | --- | --- |
| Extraversion | -0.128*** | -0.128*** | -0.128*** | -0.129*** | -0.129*** |
|  | (0.010) | (0.010) | (0.010) | (0.010) | (0.010) |
| Neuroticism | 0.140*** | 0.139*** | 0.139*** | 0.139*** | 0.141*** |
|  | (0.012) | (0.012) | (0.012) | (0.012) | (0.014) |
| Conscientiousness | 0.111*** | 0.110*** | 0.106*** | 0.110*** | 0.110*** |
|  | (0.012) | (0.012) | (0.013) | (0.012) | (0.012) |
| Openness | 0.073*** | 0.072*** | 0.073*** | 0.073*** | 0.073*** |
|  | (0.010) | (0.010) | (0.010) | (0.010) | (0.010) |
| Agreeableness | 0.252*** | 0.255*** | 0.256*** | 0.256*** | 0.255*** |
|  | (0.012) | (0.011) | (0.012) | (0.012) | (0.012) |
| Vaccination Rate | 0.001*** | 0.001*** | 0.001*** | 0.001*** | 0.001*** |
|  | (0.000) | (0.000) | (0.000) | (0.000) | (0.000) |
| Trait * Vaccination Rate | -0.002*** | -0.001*** | -0.002*** | -0.000 | 0.001*** |
|  | (0.000) | (0.000) | (0.000) | (0.000) | (0.000) |
| Constant | 0.472*** | 0.472*** | 0.472*** | 0.473*** | 0.472*** |
|  | (0.022) | (0.022) | (0.023) | (0.022) | (0.022) |
| lns1_1_1 | -3.987*** | -6.602*** | -3.621*** | -4.747*** | -3.805*** |
| _cons | (0.502) | (0.034) | (0.383) | (0.874) | (0.439) |
| lns1_1_2 | -5.094*** | -5.184*** | -5.012*** | -5.149*** | -5.035*** |
| _cons | (0.439) | (0.531) | (0.351) | (0.481) | (0.379) |
| atr1_1_1_2 | -8.557*** | -7.554 | -9.511*** | -7.868*** | 9.433*** |
| _cons | (2.929) | (4.690) | (0.695) | (1.439) | (0.624) |
| lnsig_e | -1.044*** | -1.043*** | -1.044*** | -1.043*** | -1.043*** |
| _cons | (0.017) | (0.017) | (0.017) | (0.017) | (0.017) |
| N | 32277 | 32277 | 32277 | 32277 | 32277 |

Note: Controls for education, income, age, gender, and province; cluster robust standard errors in parentheses; * p<0.1, ** p<0.05, *** p<0.01

**Table S3.** Multi-level model estimates, COVID-19 caseload

|  | Agreeableness | Openness | Conscientiousness | Extraversion | Emotional instability |
| --- | --- | --- | --- | --- | --- |
| Extraversion | -0.128*** | -0.128*** | -0.128*** | -0.128*** | -0.128*** |
|  | (0.010) | (0.010) | (0.010) | (0.010) | (0.010) |
| Neuroticism | 0.139*** | 0.139*** | 0.139*** | 0.139*** | 0.141*** |
|  | (0.012) | (0.012) | (0.012) | (0.012) | (0.015) |
| Conscientiousness | 0.111*** | 0.110*** | 0.107*** | 0.110*** | 0.110*** |
|  | (0.012) | (0.012) | (0.013) | (0.012) | (0.012) |
| Openness | 0.073*** | 0.072*** | 0.073*** | 0.073*** | 0.073*** |
|  | (0.010) | (0.010) | (0.010) | (0.010) | (0.010) |
| Agreeableness | 0.253*** | 0.255*** | 0.256*** | 0.256*** | 0.256*** |
|  | (0.011) | (0.011) | (0.012) | (0.012) | (0.012) |
| Vaccination Rate | 0.001*** | 0.001*** | 0.001*** | 0.001*** | 0.001*** |
|  | (0.000) | (0.000) | (0.000) | (0.000) | (0.000) |
| Trait * Vaccination Rate | -0.003*** | -0.001*** | -0.002*** | -0.000 | 0.001*** |
|  | (0.000) | (0.000) | (0.000) | (0.000) | (0.000) |
| Caseload | 0.000** | 0.000** | 0.000** | 0.000** | 0.000** |
|  | (0.000) | (0.000) | (0.000) | (0.000) | (0.000) |
| Trait * Caseload | -0.000*** | -0.000 | -0.000*** | -0.000 | 0.000 |
|  | (0.000) | (0.000) | (0.000) | (0.000) | (0.000) |
| Constant | 0.472*** | 0.472*** | 0.472*** | 0.472*** | 0.472*** |
|  | (0.023) | (0.023) | (0.023) | (0.023) | (0.023) |
| lns1_1_1 | -15.835*** | -17.406 | -20.574 | -15.463*** | -8.923*** |
| _cons | (1.576) | (48.902) | (73.374) | (1.571) | (0.038) |
| lns1_1_2 | -5.481*** | -5.470*** | -5.485*** | -5.476*** | -5.475*** |
| _cons | (0.719) | (0.709) | (0.724) | (0.714) | (0.710) |
| lnsig_e | -1.044*** | -1.043*** | -1.043*** | -1.043*** | -1.043*** |
| _cons | (0.017) | (0.017) | (0.017) | (0.017) | (0.017) |
| N | 32277 | 32277 | 32277 | 32277 | 32277 |

Note: Controls for education, income, age, gender, and province; cluster robust standard errors in parentheses; * p<0.1, ** p<0.05, *** p<0.01. Models estimated with an independent variance-covariance structure.

# Supplementary Figures

**
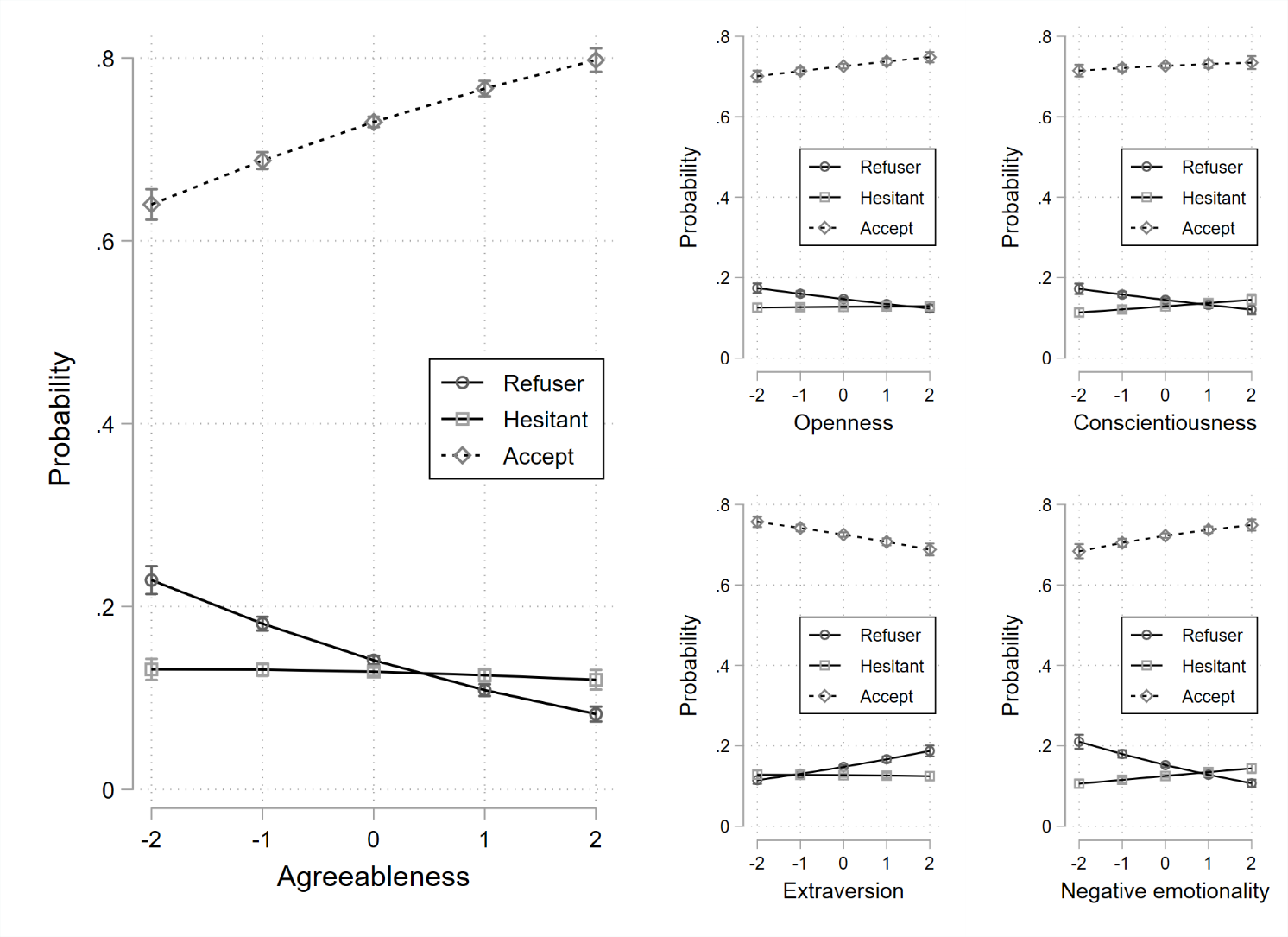
**

**Figure S1.** Predicted probabilities from multinomial logistic regression estimation. Agreeableness (left); Openness (top-center); Conscientiousness (top-right); Extraversion (bottom-center); Negative emotionality (bottom-right). 95% confidence intervals.
